# The determinants of growth failure in children under five in 25 low- and middle-income countries

**DOI:** 10.7189/jogh.13.04077

**Published:** 2023-08-04

**Authors:** Stephen Jiang, Jerry Sung, Rakshat Sawhney, Jinxuan Cai, Huaying Xu, Shu Kay Ng, Jing Sun

**Affiliations:** 1 School of Medicine and Dentistry, Griffith University, Gold Coast, Queensland, Australia; 2 Menzies Health Institute Queensland, Griffith University, Gold Coast, Queensland, Australia

## Abstract

**Background:**

Past studies have identified determinants of growth failure (GF) such as socio-economic, nutritional, parenting, and inequality factors. However, few studies investigate the numerous causes of GF across multiple countries. By analysing the data of children under five in 25 low and middle-income countries, this study aims to examine the correlations of determinants with GF to identify the strongest modifiable risk factors.

**Methods:**

Cross-sectional study design was used, and data were collected across 25 LMICs by the United Nations Children's Fund in 2019. Regions and households were randomly selected in participating LMICs. The four outcome measures were stunting, wasting, underweight and low body mass index (BMI).

**Results:**

Multilevel analysis was performed to identify the impact of country, suburb, and household levels on the variance of outcome variables. GF measures were significantly correlated with low gross domestic product (GDP) per capita (odds ratio (OR) = 2.482), rural areas (OR = 1.223), lack of health insurance (OR = 1.474), low maternal education (OR = 2.260), lack of plain water (OR = 1.402), poor maternal physical caregiving ability (OR = 1.112), low carbohydrate consumption (OR = 1.470), and continued breastfeeding in children >12 months old (OR = 0.802).

**Conclusions:**

By identifying key GF risk factors, this study may provide valuable insights for policymaking and interventions. This may allow the prioritisation of resources within countries for preventative measures to be developed.

Despite improvements in rates of growth failure (GF) since 2000, one in four children under five in low- and middle-income countries (LMICs) still suffered from at least one dimension of GF in 2020 [1]. Global estimates of GF rates determined that 22% of children were stunted and 7% were wasted in 2020, with 85% of GF occurring in LMICs [2-6]. GF manifests as anthropometric failures characterised by insufficient height or weight in reference to age-specific growth standards [1]. Assessment of anthropometric failure is traditionally done with the indicators of stunting, wasting, and underweight, which are defined as height-for-age, weight-for-height, and weight-for-age respectively that are more than two standard deviations below the World Health Organisation's (WHO) median child growth reference standards [1]. Children living in LMICs have faced many grave challenges due to socioeconomic disadvantages, including extreme poverty, inadequate access to health care services, food insecurity and poor nutrition [7-11]. Furthermore, household factors such as maternal education and caregiving ability can impact feeding practices, rates of breastfeeding, and the overall burden of child anthropometric failure, which will also be addressed in this study [12,13].

A major gap in current research is the lack of studies that examine multiple determinants across various LMICs. Prior research on GF tends to focus on and assess a single factor or a small subset of factors, while other studies only investigated one country such as Ethiopia or Bangladesh [13-20]. Consequently, there is a lack of studies that assess cross-country heterogeneity and the relative significance of different determinants at the household, region, and country levels [13]. It is hypothesized determinants in all three of these levels will demonstrate a correlation with the burden of GF amongst children under five. This study aims to determine the prevalence of GF measures across multiple LMICs to identify countries that are most severely affected and examine the correlations of determinants with GF to identify the strongest modifiable risk factors.

## METHODS

This multivariable secondary data analysis study investigated 25 different LMICs and the correlations of various socioeconomic and household factors with GF in children under five.

### Data source

The data was collected through Multiple Indicator Cluster Surveys (MICS) by UNICEF. MICS is an international household survey program developed by UNICEF in the 1990s to obtain statistically sound and internationally comparable data on a wide range of indicators regarding the situation of men and women [21]. This study analysed the 2019 surveys of mothers with children under five of both sexes from 25 LMICs. This included Mongolia, Bangladesh, Nepal, Iraq, Kiribati, Zimbabwe, Serbia, Algeria, Central African Republic, Chad, Costa Rica, Cuba, Georgia, Guinea Bissau, Kosovo, Kyrgyzstan, Lesotho, Montenegro, Republic of North Macedonia, Palestine, Suriname, Gambia, Tonga, Tunisia, and Turkmenistan. The sampling frame was based on postcode address files and cluster sampling was used. The data was collected via face-to-face interviews from randomly selected households in randomly selected suburbs for each country for a total of 173 365 participants. All MICS surveys are based on representative samples, selected by using probabilistic, random samples. The provinces within each country were identified. The principal strata for sampling were determined to be the urban and rural areas within each province. A predetermined number of suburbs within each stratum were deliberately chosen at the first sampling step with probability proportionate to size. Within the chosen suburbs, a household listing was done to determine which families had and did not have children under the age of five. Through a computer-based systematic random selection procedure, households with children under the age of five were chosen in each sample enumeration region. If an interview was refused in a selected household, the supervisor of the team returned to that household to explain the importance of the survey and to encourage the respondent to participate. If the household still refused to be interviewed, the result of the household interview was marked as “refused”.

Permission from the UNICEF office was provided to the principal supervisor and all collaborators to use the data, so no ethics application was required. All data were de-identified when provided, allowing confidentiality to be maintained. Approval forms were signed to receive permission from each country to access the participant information.

#### Outcome variables

The outcome variables were used to determine GF and included body mass index (BMI) for age, height for age, weight for height, and weight for age. The age and sex were recorded to assist in assessing the number of standard deviations from the mean for each child with reference to the WHO growth charts Z score tables “Birth to 2 years” and “2 to 5 years” [22].

#### Predictors

The three levels of data were country, region, and individual / household. The individual-level independent variables were nutrition, breastfeeding, caregiving ability and inequalities (wealth index, health insurance, fluid intake and mother's education). Region-level factors included area (urban, rural), and suburb. Country-level factors included GDP per capita and continent.

#### Nutrition

Mothers were asked whether their children ate certain types of food yesterday such as mangoes and papayas. Vegetables included pumpkin, carrots, squash etc. that are yellow or orange inside, as well as green leafy vegetables. Carbohydrates included foods made from grains and those made from roots such as white potatoes, white yams, manioc, cassava etc. The eight protein variables included eggs, animal milk, yoghurt, meat, organ meat such as liver, fish or shellfish, beans, and cheese.

#### Breastfeeding

Breastfeeding status was determined by asking if the child is currently being breastfed and if the child has ever been breastfed in the past. To further examine the effects of breastfeeding in different age groups and the impacts of extended breastfeeding, separate logistic regression analyses have been performed for breastfeeding in children above and below the age of 12 months.

#### Caregiving ability

Both the physical and emotional caregiving data of mothers were collected, which were merged to create physical and emotional caregiving scores. The Cronbach's alpha values for physical and emotional caregiving variables were 0.665 and 0.506 respectively. Physical caregiving ability was determined by the level of physical abuse / punishment and emotional caregiving ability was determined by levels of shouting and verbal abuse. Emotional caregiving includes factors such as whether mothers yelled at children or called them stupid, while physical caregiving describes the body part and mechanism by which children were hit by their mothers.

#### Inequalities

Inequalities included wealth index, health insurance status, liquid and supplement intake, mother’s education level, and whether the household was in a rural or urban area. Wealth index tercile measurement produced three groups ranging from lowest wealth to highest. Liquid intake included whether children drank various types of liquids yesterday such as oral rehydration solution (ORS), supplements, and plain water. Mother’s education was recorded as three groups: First is attended primary school or no education, second is attended secondary school and third is attended high school or above.

#### Statistical analysis

Data from the 25 countries were merged into one SPSS file while ensuring that the variables obtained in different countries are comparable. Only variables that were present in the data files of all 25 countries were kept in this study. G*Power software was used to calculate the required sample size for the study. A priori power analysis was utilised with 5% type 1 error and power of 80%. The minimum two-tailed sample size required was calculated to be 22521 participants after adjusting for non-response rate, observational study design, and cluster sampling.

χ^2^ analyses were performed to compare the BMI distribution of children under five across the 25 low-income countries. The percentages of people in various BMI categories were obtained for each country. This allowed the identification of countries that are suffering the most from GF.

As all independent and dependent variables are categorical, χ^2^ analyses were performed to investigate the correlation of nutrition, breastfeeding, caregiving ability, and inequalities with GF.

Logistic regression analysis evaluated the prediction of growth failure by determinants when gender and age were controlled in the analysis. Outcome measures were recoded into dichotomous variables (stunted, wasted, underweight, BMI less than -2 standard deviation (SD)).

Given that the sample size is large and that there are three levels of data, multilevel analysis was performed to identify the impact that country, suburb, and household levels have on the variance of outcome variables. Multilevel models (MLM) can account for the inherent hierarchal structure of the data and contextual influences. STATA version 16.0 (StataCorp, College Station, TX) was purchased and used to conduct the multilevel analysis. A significance level of 0.05 was used and all analyses were performed on SPSS.

## RESULTS

Overall, data was collected from 173 365 participants from 25 countries, with sample sizes per country ranging from 1329 in Montenegro to 24 686 in Bangladesh.

### Prevalence of growth failure measures across countries

**Figure 1:**
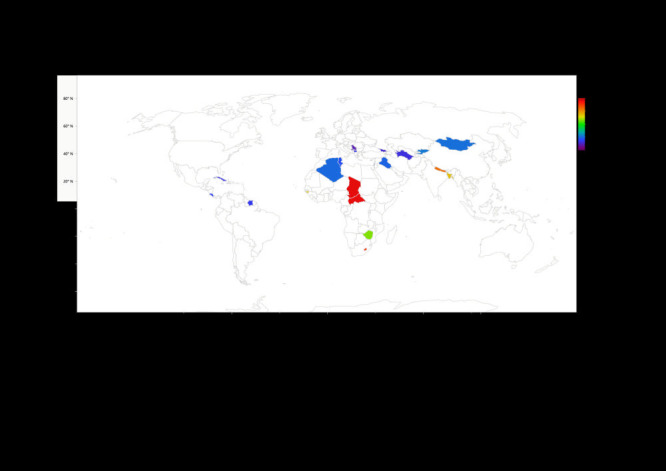
Percentage of stunted children across 25 low- and middle-income countries (LMICs).

**Figure 2:**
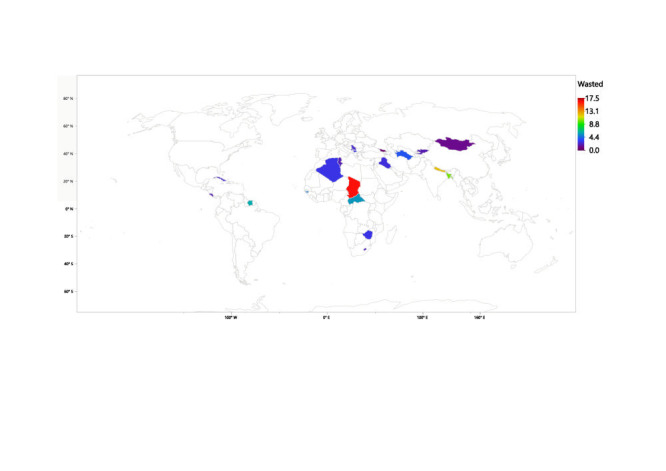
Percentage of wasted children across 25 low- and middle-income countries (LMICs).

**Figure 3:**
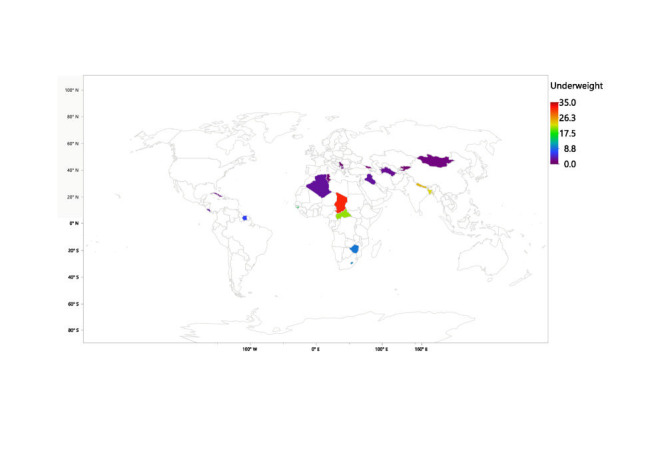
Percentage of underweight children across 25 low- and middle-income countries (LMICs).

**Figure 4:**
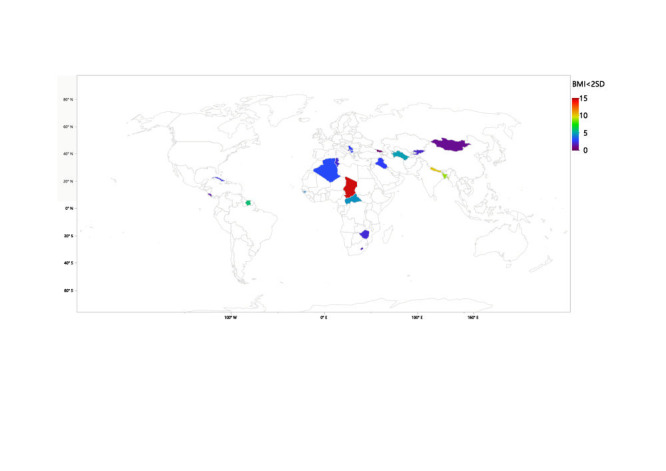
Percentage of children with low body mass index (BMI) (below -2 standard deviation (SD)) across 25 low- and middle-income countries (LMICs).

**Table 1** shows the prevalence of the GF measures under consideration across the various countries. Overall, compared with low-income countries, upper-middle-income countries experienced significantly less (*P* < 0.001) stunting (6.47% vs. 31.8%), wasting (2.73% vs. 8.45%), underweight (2.72% vs. 20.98%) and low BMI (2.84% vs. 7.38%). Chad had the highest prevalence of all four GF factors studied in this paper. Chad had the highest prevalence of stunting (8253, 38.6%), closely followed by the Central African Republic (3309, 38.5%), and Lesotho (1113, 35.5%). The three countries with the highest prevalence of stunting are all from Africa. Apart from stunting, the two countries following Chad in all three of the other GF measures being studied were Nepal and Bangladesh in that order. This indicates a high prevalence of GF in the low-income countries of Asia. Tonga, Georgia, and Serbia had some of the lowest values for prevalence across the four measures being studied. **Figure 1**, **Figure 2**, **Figure 3** and **Figure 4** provide visual maps showcasing the distribution of GF measures across the 25 LMICs.

**Table 1:** Prevalence of growth failure measures across countries

Country / income category	Stunted n (%)		Country / income category	Wasted n (%)		Country / income category	Underweight n (%)		Country / income category	BMI<-2 SD n (%)	
Low	15 622	(31.80%)	Low	5013	(8.45%)	Low	11 420	(20.98%)	Low	4413	(7.38%)
Low middle	14 386	(18.50%)	Low middle	4050	(4.09%)	Low middle	8689	(8.56%)	Low middle	3691	(3.74%)
Upper middle	3258	(6.47%)	Upper middle	1186	(2.73%)	Upper middle	1291	(2.72%)	Upper middle	1242	(2.84%)
**Low-income countries**											
Chad	8253	(38.6%)	Chad	3505	(16.5%)	Chad	6917	(32.0%)	Chad	3118	(14.7%)
Central African Republic	3309	(38.5%)	The Gambia	661	(6.8%)	Central African Republic	1836	(21.0%)	The Gambia	568	(5.8%)
Guinea Bissau	2013	(27.3%)	Central African Republic	486	(5.6%)	The Gambia	1520	(15.5%)	Central African Republic	407	(4.7%)
The Gambia	2047	(21.1%)	Guinea Bissau	361	(4.9%)	Guinea Bissau	1147	(15.4%)	Guinea Bissau	320	(4.3%)
**Low middle-income countries**											
Lesotho	1113	(35.5%)	Nepal	792	(12.2%)	Nepal	1639	(24.8%)	Nepal	672	(10.3%)
Nepal	2112	(32.6%)	Bangladesh	2219	(10.1%)	Bangladesh	5185	(23.1%)	Bangladesh	1992	(9.0%)
Bangladesh	6174	(27.9%)	Kiribati	76	(3.5%)	Lesotho	329	(10.4%)	Kiribati	74	(3.5%)
Zimbabwe	1401	(23.5%)	Algeria	418	(3.0%)	Zimbabwe	581	(9.7%)	Algeria	443	(3.2%)
Kiribati	334	(15.7%)	Zimbabwe	176	(3.0%)	Kiribati	153	(7.1%)	Tunisia	75	(2.3%)
Kyrgyz Republic	384	(11.2%)	Lesotho	68	(2.2%)	Algeria	441	(3.1%)	Zimbabwe	140	(2.3%)
Mongolia	633	(10.7%)	Tunisia	68	(2.1%)	State of Palestine	127	(2.2%)	Kyrgyz Republic	72	(2.1%)
Algeria	1441	(10.3%)	Kyrgyz Republic	70	(2.0%)	Mongolia	126	(2.1%)	Lesotho	57	(1.8%)
State of Palestine	503	(8.8%)	State of Palestine	85	(1.5%)	Tunisia	55	(1.6%)	State of Palestine	95	(1.7%)
Tunisia	291	(8.8%)	Mongolia	78	(1.3%)	Kyrgyz Republic	53	(1.5%)	Mongolia	71	(1.2%)
**Upper middle-income countries**											
Iraq	1640	(10.0%)	Suriname	207	(6.1%)	Suriname	235	(6.7%)	Suriname	202	(6.0%)
Costa Rica	252	(7.9%)	Turkmenistan	145	(4.0%)	Kosovo	72	(3.7%)	Turkmenistan	182	(5.0%)
Kosovo	139	(7.4%)	Republic of North Macedonia	58	(3.1%)	Costa Rica	103	(3.2%)	Republic of North Macedonia	60	(3.2%)
Suriname	247	(7.3%)	Iraq	457	(2.8%)	Iraq	529	(3.2%)	Serbia	34	(2.9%)
Cuba	366	(7.0%)	Montenegro	20	(2.6%)	Turkmenistan	103	(2.8%)	Iraq	479	(2.9%)
Republic of North Macedonia	127	(6.8%)	Kosovo	48	(2.6%)	Republic of North Macedonia	48	(2.5%)	Kosovo	50	(2.7%)
Turkmenistan	236	(6.5%)	Serbia	29	(2.5%)	Cuba	123	(2.3%)	Cuba	137	(2.6%)
Georgia	118	(5.8%)	Cuba	130	(2.5%)	Georgia	38	(1.8%)	Montenegro	17	(2.2%)
Montenegro	38	(4.8%)	Costa Rica	66	(2.1%)	Montenegro	13	(1.6%)	Tonga	20	(1.6%)
Serbia	55	(4.6%)	Tonga	16	(1.2%)	Tonga	16	(1.2%)	Costa Rica	50	(1.6%)
Tonga	40	(3.1%)	Georgia	10	(0.5%)	Serbia	11	(0.9%)	Georgia	11	(0.5%)
**Total**	33266	(20.8%)	Total	10249	(6.4%)	Total	21400	(13.2%)	Total	9346	(5.9%)
**Chi Square (χ2)**	14072.871*		Chi Square (χ2)	6627.020*		Chi Square (χ2)	17104.816*	Chi Square (χ2)		5378.702*	

BMI – body mass index, SD – standard deviation

*P < 0.001

#### Prevalence of key growth failure determinants across countries

**Table 2** shows the prevalence of low maternal education, no health insurance, no carbohydrate consumption, and no plain water consumption across the various countries. Overall, compared with upper-middle-income countries, low-income countries had more mothers with only primary or no education (86.3% vs. 46.6%), more households with no health insurance (99.5% vs. 57.7%), and more children who did not consume carbohydrates (49.2% vs. 29.3%) and plain water (23.2% vs. 16.0%) yesterday. These results were all statistically significant with a *P* < 0.001. Chad, Central African Republic, and Guinea Bissau had the highest prevalence of unfavourable determinants. Overall, these results indicate that countries with higher incomes generally had better determinant measures which can potentially improve the health of children.

**Table 2:** Prevalence of key growth failure determinants across countries

Country / income category	Mother’s education – primary or none n (%)		Country / income category	Without health insurance n (%)		Country / income category	No carbohydrate consumption yesterday n (%)		Country / income category	No plain water consumption yesterday n (%)	
Low	41 658	(86.3%)	Low	47 791	(99.5%)	Low	9371	(49.2%)	Low	4682	(23.2%)
Low middle	20 255	(33.1%)	Low middle	22 304	(59.8%)	Low middle	9506	(29.6%)	Low middle	8220	(28.2%)
Upper middle	19 431	(46.6%)	Upper middle	22 124	(57.7%)	Upper middle	4890	(29.3%)	Upper middle	2663	(16.0%)
**Low-income countries**											
Guinea Bissau	7058	(94.3%)	Chad	21 730	(99.8%)	Chad	6917	(32.0%)	Chad	3118	(14.7%)
Chad	19 705	(90.1%)	Central African Republic	8849	(99.4%)	Central African Republic	18 36	(21.0%)	The Gambia	568	(5.8%)
Central African Republic	7262	(80.4%)	Guinea Bissau	7412	(99.3%)	The Gambia	1520	(15.5%)	Central African Republic	407	(4.7%)
The Gambia	7633	(77.2%)	The Gambia	9800	(99.0%)	Guinea Bissau	1147	(15.4%)	Guinea Bissau	320	(4.3%)
**Low middle-income countries**											
Lesotho	1659	(51.0%)	Nepal	6361	(95.6%)	Nepal	1639	(24.8%)	Nepal	672	(10.3%)
Tunisia	1105	(44.6%)	Zimbabwe	5739	(94.1%)	Bangladesh	5185	(23.1%)	Bangladesh	1992	(9.0%)
Algeria	4823	(37.7%)	Algeria	7803	(52.6%)	Lesotho	329	(10.4%)	Kiribati	74	(3.5%)
Bangladesh	8543	(34.6%)	Palestine	1825	(28.8%)	Zimbabwe	581	(9.7%)	Algeria	443	(3.2%)
Zimbabwe	2063	(33.8%)	Tunisia	576	(16.9%)	Kiribati	153	(7.1%)	Tunisia	75	(2.3%)
Mongolia	337	(20.9%)				Algeria	441	(3.1%)	Zimbabwe	140	(2.3%)
Kiribati	448	(20.5%)				State of Palestine	127	(2.2%)	Kyrgyz Republic	72	(2.1%)
State of Palestine	1269	(19.8%)				Mongolia	126	(2.1%)	Lesotho	57	(1.8%)
Kyrgyz Republic	8	(0.5%)				Tunisia	55	(1.6%)	State of Palestine	95	(1.7%)
						Kyrgyz Republic	53	(1.5%)	Mongolia	71	(1.2%)
**Upper middle-income countries**											
Turkmenistan	2989	(85.2%)	Iraq	16 522	(99.4%)	Suriname	235	(6.7%)	Suriname	202	(6.0%)
Iraq	10 952	(65.9%)	Kosovo	2182	(96.0%)	Kosovo	72	(3.7%)	Turkmenistan	182	(5.0%)
Montenegro	741	(64.9%)	Turkmenistan	2099	(57.0%)	Costa Rica	103	(3.2%)	Republic of North Macedonia	60	(3.2%)
Kosovo	1324	(64.8%)	Tonga	248	(18.4%)	Iraq	529	(3.2%)	Serbia	34	(2.9%)
Republic of North Macedonia	904	(39.9%)	Suriname	495	(11.7%)	Turkmenistan	103	(2.8%)	Iraq	479	(2.9%)
Suriname	1011	(27.3%)	Costa Rica	351	(9.7%)	Republic of North Macedonia	48	(2.5%)	Kosovo	50	(2.7%)
Costa Rica	943	(26.1%)	Republic of North Macedonia	114	(5.2%)	Cuba	123	(2.3%)	Cuba	137	(2.6%)
Georgia	287	(18.5%)	Georgia	89	(3.5%)	Georgia	38	(1.8%)	Montenegro	17	(2.2%)
Serbia	175	(8.9%)	Serbia	24	(1.3%)	Montenegro	13	(1.6%)	Tonga	20	(1.6%)
Tonga	36	(2.6%)				Tonga	16	(1.2%)	Costa Rica	50	(1.6%)
Cuba	69	(1.8%)				Serbia	11	(0.9%)	Georgia	11	(0.5%)
**Total**	81 344	(53.8%)	Total	92 219	(74.6%)	Total	21 400	(13.2%)	Total	9346	(5.9%)
**Chi Square (χ2)**	68187.371*		Chi Square (χ2)	78318.305*		Chi Square (χ2)	17104.816*	Chi Square (χ2)		5378.702*	

*P < 0.001.

#### Correlations of determinants with growth failure

**Table 3** presents correlations of child and family characteristics with stunting, wasting, underweight, and BMI less than 2SD. All GF measures were significantly (*P* < 0.05) more prevalent for boys, the poorest wealth quintile, no health insurance, and rural areas. Overall, most GF measures were increased in groups that consumed less food, including those who on the previous day did not eat grains or roots, protein, yellow or orange vegetables and vitamin A-rich fruits. However, those who ate green leafy vegetables yesterday had significantly (*P* < 0.05) increased levels of stunting, wasting, and being underweight. Children who have ever been breastfed in the past were significantly (*P* < 0.05) less stunted and underweight, while those older than 12 months who are still currently being breastfed had significantly (*P* < 0.05) higher rates of wasting, underweight, and low BMI. The age group with the most stunted and underweight children was the 2-3 years old group, while the 0-1 group had the most wasted and low BMI children. Mothers who had the lowest education (primary or none), poor physical caregiving ability and did not explain to the children why their behaviour was wrong had significantly (*P* < 0.05) increased levels of all GF measures.

**Table 3:** Associations of determinants with growth failure

Variable	Stunted n (%)	Not stunted n (%)	Wasted n (%)	Not wasted n (%)	Underweight n (%)	Not underweight n (%)	BMI≤-2 SD n (%)	-1.99 SD to normal BMI n (%)
**Sex**						
Male	17 654* (21.6%)	64 185 (78.4%)	5680* (7.0%)	76 038 (93.0%)	11 397* (13.7%)	71 563 (86.3%)	5023* (6.2%)	76 613 (93.8%)
Female	15 612 (20.0%)	62 626 (80.0%)	4569 (5.9%)	73 501 (94.1%)	10 003 (12.6%)	69 192 (87.4%)	4323 (5.5%)	73 737 (94.5%)
**Age of child**								
0-1	3751* (12.5%)	26 171 (87.5%)	2581* (8.6%)	27 268 (91.4%)	3347* (11.0%)	27 217 (89.0%)	2811* (9.4%)	27 233 (90.6%)
1-2	6978 (22.9%)	23 528 (77.1%)	2177 (7.1%)	28 402 (92.9%)	4178 (13.5%)	26 882 (86.5%)	1789 (5.9%)	28 696 (94.1%)
2-3	8422 (26.4%)	23426 (73.6%)	1865 (5.9%)	29910 (94.1%)	4857 (15.0%)	27 514 (85.0%)	1574 (5.0%)	30 114 (95.0%)
3-4	7870 (23.0%)	26366 (77.0%)	1739 (5.1%)	32398 (94.9%)	4633 (13.4%)	29 834 (86.6%)	1516 (4.5%)	32 517 (95.5%)
4-5	6245 (18.6%)	27320 (81.4%)	1887 (5.6%)	31561 (94.4%)	4385 (13.0%)	29 308 (87.0%)	1656 (5.0%)	31 790 (95.0%)
**Wealth index tercile**								
Poorest	18 324* (23.9%)	58 191 (76.1%)	5246* (6.9%)	71 191 (93.1%)	5492* (10.4%)	47 408 (89.6%)	2907* (5.6%)	49 087 (94.4%)
Middle	6358 (20.2%)	25 060 (79.8%)	1932 (6.2%)	29 416 (93.8%)	4095 (12.9%)	27 714 (87.1%)	1763 (5.6%)	29 576 (94.4%)
Richest	8584 (16.5%)	43 560 (83.5%)	3071 (5.9%)	48 932 (94.1%)	11 823 (15.3%)	65 633 (84.7%)	4676 (6.1%)	71 687 (93.9%)
**Health insurance**						
Without	22 019* (24.7%)	67 296 (75.3%)	6802* (7.6%)	82 429 (92.4%)	14 526* (16.1%)	75 806 (83.9%)	6089* (6.8%)	83 073 (93.2%)
With	2168 (7.8%)	25 678 (92.2%)	775 (2.8%)	26 960 (97.2%)	864 (3.0%)	27 519 (97.0%)	827 (3.0%)	26 867 (97.0%)
**Mother’s education**							
Primary or none	20 988* (27.0%)	56 692 (73.0%)	6295* (8.1%)	71 303 (91.9%)	14 164* (18.0%)	64 327 (82.0%)	5629* (7.3%)	71 888 (92.7%)
Secondary	6753 (17.7%)	31 344 (82.3%)	2086 (5.5%)	35 937 (94.5%)	4253 (11.0%)	34 429 (89.0%)	1930 (5.1%)	36 078 (94.9%)
Higher	2471 (9.7%)	23 043 (90.3%)	824 (3.2%)	24 593 (96.8%)	1092 (4.2%)	24 797 (95.8%)	854 (3.4%)	24 564 (96.6%)
**Area**						
Rural	23 997* (25.5%)	69 960 (74.5%)	7111* (7.6%)	86 753 (92.4%)	16 040* (16.9%)	79 043 (83.1%)	6327* (6.7%)	87 488 (93.3%)
Urban	9143 (14.1%)	55 622 (85.9%)	3115 (4.8%)	61 468 (95.2%)	5317 (8.1%)	60 378 (91.9%)	2992 (4.6%)	61 550 (95.4%)
**Child drank plain water yesterday**						
No	2640* (17.9%)	12 116 (82.1%)	1371* (9.3%)	13 319 (90.7%)	2093* (13.9%)	12 996 (86.1%)	1376* (9.3%)	13 439 (90.7%)
Yes	9094 (18.9%)	38 914 (81.1%)	3636 (7.6%)	44 442 (92.4%)	6091 (12.5%)	42 795 (87.5%)	3416 (7.1%)	44 636 (92.9%)
**Child drank any other liquid yesterday**								
No	10 124* (18.5%)	44 597 (81.5%)	4371 (8.0%)	50 350 (92.0%)	7100 (12.7%)	48 691 (87.3%)	4191 (7.6%)	50 641 (92.4%)
Yes	1593 (20.1%)	6336 (79.9%)	624 (7.9%)	7304 (92.1%)	1072 (13.3%)	6994 (86.7%)	584 (7.4%)	7330 (92.6%)
**Physical caregiving**			
Poor (score 3 and below)	7688* (25.6%)	22 293 (74.4%)	1903* (6.4%)	27 993 (93.6%)	4883* (16.2%)	25 342 (83.8%)	1598* (5.4%)	28 256 (94.6%)
Middle (score 4 to 5)	11 949* (22.5%)	41 130 (77.5%)	3014 (5.7%)	49 955 (94.3%)	7278 (13.6%)	46 302 (86.4%)	2598 (4.9%)	50 249 (95.1%)
Good (score 6)	9657* (20.9%)	36 600(79.1%)	2694 (5.8%)	43 542 (94.2%)	5750 (12.2%)	41 190 (87.8%)	2289 (5.0%)	43 824 (95.0%)
**Explained why behaviour was wrong**								
No	10 119* (27.3%)	26 883 (72.7%)	2777* (7.5%)	34 220 (92.5%)	6451 *(17.2%)	31 043 (82.8%)	2284* (6.2%)	34 622 (93.8%)
Yes	19 355 (20.8%)	73 644 (79.2%)	4877 (5.3%)	87 912 (94.7%)	11 578 (12.3%)	82 363 (87.7%)	4237 (4.6%)	88 356 (95.4%)
**Carbohydrate consumption yesterday**						
Ate none	3860* (17.2%)	18 633 (82.8%)	2249* (10.1%)	20 112 (89.9%)	3242* (14.1%)	19 736 (85.9%)	2333* (10.3%)	20 216 (89.7%)
Ate one of grains or roots	4714 (19.9%)	18 992 (80.1%)	1680 (7.1%)	22 095 (92.9%)	2986 (12.4%)	21 104 (87.6%)	1489 (6.3%)	22 221 (93.7%)
Ate both grains and roots	2920 (16.1%)	15 229 (83.9%)	940 (5.2%)	17 267 (94.8%)	1607 (8.7%)	16 915 (91.3%)	887 (4.9%)	17 288 (95.1%)
**Child ate green leafy vegetables yesterday**						
No	8555* (16.6%)	42 872 (83.4%)	3826* (7.4%)	47 550 (92.6%)	5926* (11.3%)	46 507 (88.7%)	3771 (7.3%)	47 732 (92.7%)
Yes	2949 (22.7%)	10 026 (77.3%)	1049 (8.1%)	11 973 (91.9%)	1921 (14.5%)	11 292 (85.5%)	942 (7.3%)	12 044 (92.7%)
**Child ever been breastfed**						
No	1756* (23.7%)	5652 (76.3%)	510 (6.9%)	6892 (93.1%)	1079* (14.3%)	6479 (85.7%)	459 (6.2%)	6930 (93.8%)
Yes	17 367 (20.5%)	67 326 (79.5%)	6107 (7.2%)	78 521 (92.8%)	11 292 (13.1%)	74 969 (86.9%)	5710 (6.7%)	78 947 (93.3%)
**Child still being breastfed**		
No	7370 (20.7%)	28 212 (79.3%)	1612* (4.5%)	33 920 (95.5%)	3658* (10.1%)	32 511 (89.9%)	1472* (4.2%)	33 977 (95.8%)
Yes	9991 (20.4%)	39 088 (79.6%)	4491 (9.2%)	44 573 (90.8%)	7630 (15.2%)	42 429 (84.8%)	4234 (8.6%)	44 942 (91.4%)

BMI – body mass index, SD – standard deviation

*Indicates significant χ^2^ result (*P* < 0.05) for the variable.

We also investigated several variables which were not significantly correlated with any GF outcomes in the **Table 4** logistic regression analysis and were not displayed in both **Table 3** and **Table 4** to ensure clarity. These variables included the consumption of ORS, vitamin or mineral supplements, proteins such as eggs, meat and dairy, vegetables such as pumpkin, carrots squash etc. that are yellow or orange inside yesterday, ripe mangoes, papayas etc. any other vitamin A-rich fruits, infant formula, fortified baby food and mother's emotional caregiving ability.

**Table 4:** Prediction of growth failure by determinants when gender and age are controlled in the analysis

Variables	OR (95% CI) stunting (HAZ<-2 SD)		OR (95% CI) wasting (WHZ<-2 SD)		OR (95% CI) underweight (WAZ<-2 SD)		OR (95% CI) BMI<-2 SD	
**GDP per capita**								
4391.34 and above	1		1		1		1	
1855.75 to 4391.33	1.472*	(1.171-1.851)	0.736	(0.419-1.292)	1.181	(0.725-1.925)	0.671	(0.406-1.106)
777.82 to 1855.74	4.217*	(3.080-5.773)	1.321	(0.642-2.719)	7.534*	(3.995-14.209)	0.942	(0.468-1.898)
777.81 and below	4.376*	(3.316-5.775)	3.084*	(1.602-5.939)	13.391*	(7.324-24.484)	2.482†	(1.365-4.513)
**Continent**								
Asia	1		1		1		1	
Europe	0.725‡	(0.542-0.970)	1.167	(0.644-2.114)	1.104	(0.637-1.912)	0.919	(0.535-1.577)
Americas	1.272	(0.964-1.679)	3.584*	(2.279-5.636)	3.803*	(2.480-5.832)	1.816†	(1.163-2.837)
Africa	0.806	(0.630-1.032)	1.705	(0.922-3.153)	0.791	(0.447-1.397)	1.230	(0.707-2.138)
**Area**								
Urban	1		1		1		1	
Rural	1.253*	(1.136-1.383)	1.270†	(1.074-1.502)	1.475*	(1.289-1.688)	1.223‡	(1.025-1.459)
**Wealth tercile**								
Richest	1		1		1		1	
Middle	1.124‡	(1.003-1.259)	0.863	(0.696-1.005)	0.942	(0.800-1.067)	0.881	(0.725-1.071)
Poorest	1.182†	(1.068-1.307)	0.923	(0.787-1.082)	1.057	(0.932-1.199)	0.896	(0.755-1.063)
**Health insurance**								
Yes	1		1		1		1	
No	1.213†	(1.028-1.430)	1.264	(0.906-1.763)	1.474‡	(1.077-2.016)	0.959	(0.699-1.317)
**Mother's education**								
Higher	1		1		1		1	
Secondary	1.152	(0.964-1.378)	0.976	(0.705-1.350)	1.540†	(1.107-2.141)	0.968	(0.705-1.328)
Primary or none	1.598*	(1.343-1.902)	1.421‡	(1.036-1.951)	2.260*	(1.633-3.129)	1.358	(1.000-1.844)
**Child drank plain water yesterday**								
Yes	1		1		1		1	
No	1.113	(0.986-1.257)	1.297†	(1.089-1.545)	1.313*	(1.144-1.507)	1.402*	(1.163-1.690)
**Child drank any other liquid yesterday**								
Yes	1		1		1		1	
No	0.957	(0.867-1.057)	0.829‡	(0.710-0.967)	0.824†	(0.730-0.929)	0.886	(0.749-1.047)
**Physical caregiving**	1.004	(0.970-1.040)	1.086†	(1.024-1.151)	1.104*	(1.056-1.154)	1.110†	(1.040-1.184)
**Explained why behaviour was wrong**								
Yes	1		1		1		1	
No	1.132†	(1.044-1.228)	1.164‡	(1.023-1.324)	1.204*	(1.089-1.330)	1.143	(0.994-1.314)
**Carbohydrate consumption**								
Ate both grains and roots	1		1		1		1	
Ate one	1.037	(0.940-1.144)	0.994	(0.838-1.179)	1.057	(0.926-1.208)	0.955	(0.797-1.144)
Ate none	1.320*	(1.164-1.497)	1.334†	(1.092-1.630)	1.401*	(1.197-1.640)	1.470*	(1.187-1.821)
**Child ate green leafy vegetables yesterday**								
Yes	1		1		1		1	
No	0.849*	(0.780-0.924)	0.961	(0.841-1.098)	0.910	(0.821-1.009)	0.938	(0.811-1.085)
**Child still being breastfed (age over 12 months)**								
Yes	1		1		1		1	
No	0.908‡	(0.832-0.992)	0.777*	(0.669-0.903)	0.784*	(0.698-0.880)	0.802†	(0.684-0.941)
**Child still being breastfed (age under 12 months)**								
Yes	1		1		1		1	
No	0.704	(0.431-1.150)	1.142	(0.620-2.104)	0.964	(0.526-1.767)	0.892	(0.489-1.625)
**Sex**								
Female	1		1		1		1	
Male	1.340*	(1.245-1.442)	1.372*	(1.218-1.545)	1.455*	(1.328-1.594)	1.358*	(1.193-1.545)
**Increasing age of child**	1.216‡	(1.045-1.415)	0.510*	(0.378-0.689)	0.923	(0.766-1.112)	0.529*	(0.377-0.742)
**ICC**								
Country	14.0%*	(8.5%-22.1%)			20.7%*	(13.0%-31.2%)	11.6%*	(6.9%-18.9%)
Region	18.8%*	(13.3%-26.0%)			25.3%*	(17.7%-34.8%)	20.0%*	(15.1%-25.9%)
Household	29.2%*	(24.0%-35.0%)			36.7%*	(29.8%-44.3%)	32.7%*	(27.9%-37.9%)
**Χ^2^**	2061.705*		945.983*		2664.153*		613.569*	
**R^2^**	16.3%		12.9%		24.6%		9.4%	

OR – odds ratio, CI – confidence interval, HAZ – height-for-age-z score, SD – standard deviation, WHZ – weight-for-length / height z-score, WAZ – weight-for-age-z score, BMI – body mass index, GDP – gross domestic product

*P < 0.001.

†*P* < 0.01.

‡*P* < 0.05.

**Figure 5:**
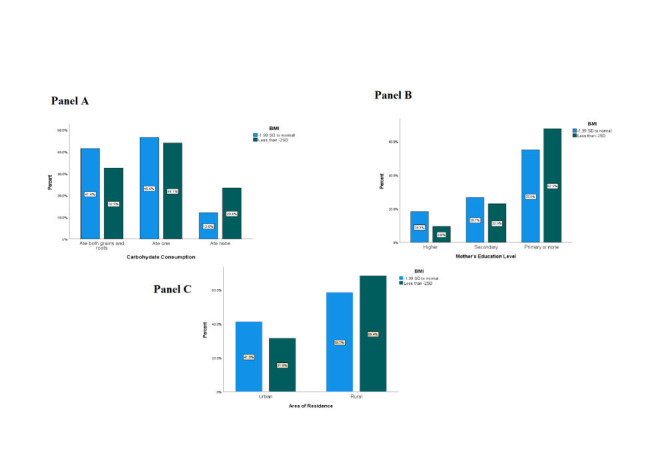
**Panel A.** Proportion of low body mass index (BMI) (below -2 standard deviation (SD)) among children with varying levels of carbohydrate consumption yesterday**. Panel B**. Proportion of low BMI (below -2 SD) among children of mothers with varying levels of education. **Panel C**. Proportion of low BMI (below -2 SD) among rural and urban residence.

**Figure 5**, panel A, panel B and panel C, showcases the proportion of low BMI (less than -2 SD) among subgroups of key determinants. It demonstrates that children of mothers with primary or no education, children who consumed fewer carbohydrates and those living in rural areas had a higher prevalence of low BMI.

#### Prediction of growth failure by determinants when gender and age are controlled in the analysis

Logistic regression analysis in **Table 4** demonstrated that GDP per capita was the strongest determinant of GF. Compared to the group with a GDP of 4391.34 and above per capita, stunting, wasting, underweight and BMI<-2 SD were 4.376, 3.084, 13.391 and 2.482 times respectively as likely to occur in the group with a GDP of 777.81 and below per capita (*P* < 0.001). All GF measures were more likely to occur in rural children compared to their urban counterparts with *P* < 0.05. Children who did not eat grains or roots yesterday were 1.320, 1.334, 1.401 and 1.470 times as likely to be stunted, wasted, underweight and have BMI<-2 SD respectively. Compared to children whose mothers attained higher education, children whose mothers attained primary or no education were significantly more likely to be underweight (*P* < 0.001), wasted (*P* < 0.05) and stunted (*P* < 0.001). All GF measures were significantly more likely to occur in boys (*P* < 0.001). Children who did not drink plain water yesterday and poor maternal physical caregiving ability were correlated with more wasting, being underweight, and BMI<-2 SD. Additionally, children without health insurance were significantly more likely to be stunted (*P* < 0.01) and underweight (*P* < 0.05).

All GF measures were significantly more likely to occur in children over 12 months old who were still being breastfed (*P* < 0.05), while there were insignificant results for children under 12 months. Children who ate green leafy vegetables yesterday were also more likely to be stunted (*P* < 0.001). Wealth tercile was not correlated with GF except children from middle-income (*P* < 0.05) and the poorest households (*P* < 0.01) were significantly more likely to be stunted than those from the wealthiest tercile within the same country. Increasing age was correlated with more stunting (*P* < 0.05) but decreased wasting (*P* < 0.001) and low BMI (*P* < 0.001). Meanwhile, protein consumption, ORS consumption, vitamin or mineral supplementation, maternal emotional caregiving ability, vegetable and fruit intake, infant formula, and fortified baby food all had little to no impact on GF.

As it is shown in **Table 4**, children in American countries (including Costa Rica and Cuba from North America, and Suriname from South America) were significantly more likely to be wasted (*P* < 0.001), underweight (*P* < 0.001), and have BMI<-2 SD (*P* < 0.01). Overall, the variables explained 16.3%, 9.4%, 24.6% and 12.9% of the total variance in stunting, BMI<-2 SD, underweight and wasting respectively.

## DISCUSSION

The four GF measures, including stunting, wasting, underweight, and BMI less than 2SD, were positively correlated with several determinants in this study. GDP per capita showed an inverse relationship with all GF measures, consistent with the impact of socioeconomic status on health [23]. Poverty can limit access to health care, contributing to increased morbidity and mortality [23]. Notably, our results show that children within the lowest GDP per capita subcategory are 13.39 times more likely to be underweight than those in the highest subcategory. This is supported by **Table 1**, which demonstrates substantially decreased GF measures in upper-middle and low-middle-income countries compared to low-income countries. Moreover, **Table 2** demonstrates that countries with higher incomes had better determinant measures, which may contribute to the decreased GF seen. However, more evidence is needed to confirm these findings due to limited representation from North and South America in this study, with only three countries in total.

In terms of regional variables, the incidence of all GF constituents was higher in rural children. Living in rural areas has been linked to limited access to health care services, a higher burden of preventable conditions, poorer financial status, lower health literacy, as well as unhealthy lifestyles such as lower levels of physical exercise and less balanced, nutritious diets [24]. Whether from their physical environment, socioeconomic status, or other social factors, rural residents are at an increased risk of adverse health outcomes [24].

Unlike factors at the country and region level, individual household factors can exert direct effects on the health and growth of children. Malnutrition is a well-established cause of GF [25]. In this study, carbohydrate consumption reduced all measures of GF. Depletion of energy stores due to a lack of sufficient carbohydrate intake can lead to GF [26]. Carbohydrate intake has been strongly linked to the growth of children, whereas the role of protein supplements in growth was shown to be inconsistent in some studies [27], which may account for the insignificant relationship between protein consumption and GF outcomes in our study. More evidence is needed to conclude the relative effectiveness of proteins and carbohydrates in preventing growth failure.

Parental factors contribute significantly to the development of GF [28]. Our study demonstrated that low maternal education levels and increased physical punishment of children were significantly correlated with GF. Higher maternal education contributed to better socioeconomic status [28] and adherence to feeding guidelines [29]. Inadequate care and family violence are increasingly recognized problems in LMICs [30]. Children from these families are more sensitive to detriments to nutrition as well as future unhealthy behaviours [30].

Our analyses also showed that breastfeeding beyond 12 months was associated with higher GF rates. Breastfeeding is correlated with numerous benefits and is therefore encouraged for newborns [31,32]. However, studies have underlined the importance of optimal breastfeeding duration and a timely introduction of a solid foods diet, as an overreliance on breastfeeding may compete with dietary diversity and lead to GF [33]. Plain water intake significantly reduced the rate of wasting, being underweight, and having low BMI. Lack of clean water access reflects lower socioeconomic status and health outcomes [34,35].

Surprisingly, the wealth index demonstrated no significant correlation with GF, except for stunting, where the poorest children are 1.18 times more likely to have stunting than the richest. Previous multi-country studies suggested moderate relationships between household wealth and GF, which may be partially explained by the differences in macroeconomic and health care systems across countries and the existence of local and national programs [36]. For example, the relationship between wealth and GF is relatively weaker in Kyrgyzstan due to investments in primary health care facilities and hospitals in disadvantaged areas [36]. Such differences may neutralize the impact of the wealth index on GF, as seen in our results. Health insurance was correlated with reduced stunting and underweight, likely due to improved access to treatment [37,38].

Data about the age and sex of children were included in the analysis as confounding factors. Boys were shown to have a significantly higher rate in all four components of GF than girls. Traditionally, social pressure focuses more on growth in boys than girls, and boys with growth abnormalities are more likely to be noticed by parents and evaluated by medical professionals [39]. A lower rate of GF would thus be expected in boys, as there is a tendency to provide more for boys in some countries [39]. However, the opposite relationship seen in our results may be explained by the differences in physiology between males and females. Males tend to have higher energy requirements than females [40,41], which can make them more prone to GF where there is limited food security and accessibility. In terms of age, older children have higher rates of stunting but lower rates of wasting and low BMI. The rate of height increase is positively correlated with age, peaking during puberty, where the velocity of height growth is around 9.5 cm (cm) per year for boys and 8.3 cm per year for girls [42]. As a result of the increasing growth speed, stunting can become prominent in older children [42]. Given that older children are more likely to develop stunting (low height for age), they are thus less likely to have wasting and low BMI since both measures are affected by height as the denominator.

Strengths of the study include a large sample size (173 365 children under five) across 25 LMICs and standardized data collection by UNICEF in 2019. Nevertheless, limitations exist in this study. First, as a cross-sectional study, inference can only be drawn on correlations instead of causality and change over time was not measured. Further studies using cohort study design or randomized controlled studies incorporating temporal data can be performed to track the progression of GF in various countries. Second, the independent variables in this study only account for about 16%, 13%, 25%, and 9% of the total variance in stunting, wasting, underweight, and low BMI, respectively. Other determinants of GF, such as father factors and the details of other siblings, can be included.

Data regarding the consumption of vegetables and fruits such as green leafy vegetables, pumpkins, and mangos, revealed little correlation with the development of GF. Despite the existence of some country-level heterogeneity, dietary diversity is shown by previous studies to have a protective effect against GF [43,44]. This is not in concordance with our results, which may be because the questions asked in this study only relate to the day before. Questionnaires can be modified to ask for long-term nutritional status instead of whether children consumed certain foods yesterday. Equally, statistics on breastfeeding duration for children are important to the scope of this study. Mothers were only asked whether their children had been breastfed before and are still breastfeeding, which limits the ability to examine relationships between breastfeeding duration and GF. There were also no objective physical and psychological examinations performed on the children, which can limit the ability to establish the adequacy of maternal caregiving. Finally, differences exist in the included countries regarding access to food and water, conflicts, and poverty. This may make it difficult to make direct comparisons between countries.

## CONCLUSIONS

Identifying key GF determinants may provide valuable insights for policymaking and interventions. This may allow the prioritization of resources within countries for preventative measures to be developed such as promoting sufficient carbohydrate intake and plain water consumption, improving access to health insurance, and enhancing maternal caregiving ability through education and health literacy. By focusing on these areas, policymakers and health care professionals can effectively address the risks of GF in children and work towards reducing the global burden of GF.
